# Primum non nocere: psychosurgery in a case of severe anorexia nervosa. A case report

**DOI:** 10.1192/j.eurpsy.2022.1531

**Published:** 2022-09-01

**Authors:** A. Cerame, M. Del Valle, P. Coucheiro Limeres, A. Franco Soler

**Affiliations:** 1 Hospital Universitario José Germain, Hospital De Día, Leganes, Spain; 2 Hospital Severo Ochoa, Psychiatry, Leganes, Spain

**Keywords:** Ethics, psychosurgery, Anorexia nervosa

## Abstract

**Introduction:**

Bilateral cingulotomy and anterior capsulotomy are two neurosurgical procedures which are reserved as a last resort for cases of severe OCD in Spain; these procedures are not approved in cases of AN.

**Objectives:**

We present the case of a 29-year-old female patient who was diagnosed with anorexia nervosa (AN) when she was 15 (2006). Due to the severity of the case the patient needed to be hospitalized for many months due to excessive weight loss. She was also treated in an out-patient department and started several intensive psychotherapeutic procedures. In 2015 the patient’s family took her to a private clinic where she was diagnosed with Obsessive-compulsive disorder (OCD) and had a bilateral cingulotomy and anterior capsulotomy.

**Methods:**

A case report where the ethical implications of the case are weighed alongside a review of the relevant literature regarding neurosurgical treatments of AN.

**Results:**

There were no significant short or long term improvements in terms of Body Mass Index or reduction of symptoms, the patient’s cognitive functions showed a decline in neuropsychological tests. Contrary to that the patient has needed hospitalizations for at least 9 months per year since the surgery and has needed admission in the Intensive Care Unit at least 3 times because of extreme malnutrition. Due to her need for chronic hospitalization was institutionalized in a long-stay psychiatric hospital.

**Conclusions:**

Psychosurgery is a controversial therapy which has limited evidence in cases of AN. Our case shows the way in which neurosurgical procedures can do more harm than good and worsen the prognosis of patients.

**Disclosure:**

No significant relationships.

